# Supplementary material for: Achillea fragrantissima (Forssk.) Sch.Bip instigates the ROS/FADD/c-PARP expression that triggers apoptosis in breast cancer cell (MCF-7)
Source: PLoS One. 2024 May 31;19(5):e0304072. doi: 10.1371/journal.pone.0304072 (PMC11142488; doi:10.1371/journal.pone.0304072)
Supplement: S1 File — (DOCX) [file pone.0304072.s001.docx]

***Achillea fragrantissima* (Forssk.) Sch.Bip instigates the ROS/FADD/c-PARP expression: An actuation of apoptosis in breast cancer cell**

**
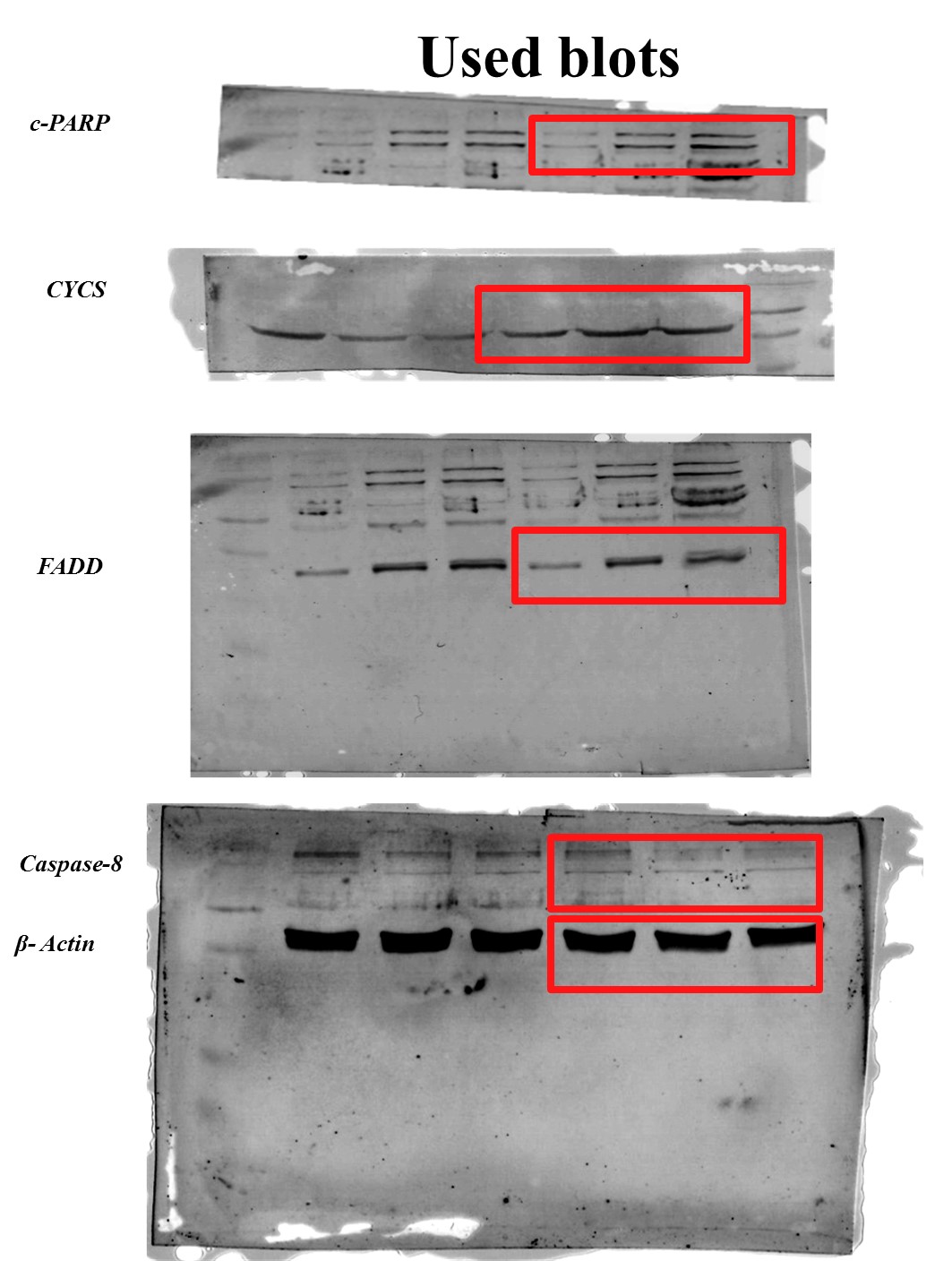
**
